# Lymphoma-Like Syndrome: 4 Case Reports About Atypical Presentation of Primary *Cytomegalovirus* Infection in Immunocompetent Children

**DOI:** 10.1097/MD.0000000000000855

**Published:** 2015-07-02

**Authors:** Marie-Gabrielle Vigué, Edouard Tuaillon, Alain Makinson, Geu Mac Bullen, Vincent Foulongne, Michel Segondy, Philippe Vande Perre, Eric Jeziorski

**Affiliations:** From the Department of Pediatric Infectious Diseases (M-GV, GMB, EJ); Montpellier University 1 (ET, PVP, EJ), Montpellier University of Medecine; Department of Bacteriology and Virology (ET, VF, MS, PVP, EJ), Montpellier University Hospital; INSERM (ET, VF, MS, PVP); Department of Infectious Diseases (AM), Montpellier University Hospital; and UMI (AM), Montpellier, France.

## Abstract

In immunocompetent persons, primary *cytomegalovirus* (CMV) infection is self-limited infection. Lymphoma-like syndromes have been sometimes described in adults but have not been described for children.

Lymphoma-like syndromes (protracted fever, alteration of the general status, and clinical lymphoproliferative syndrome) were retrospectively recorded in children attending our hospital from 1999 to 2008 for primary CMV infection. Patients with immunodeficiency, coinfection (Epstein–Barr virus, toxoplasmosis, or mycobacterial), or biological criteria of mononucleosis-like syndrome were excluded.

We report 4 cases of lymphoma-like syndrome. The median duration of fever was 21.5 days (range 15–27). Tonsillitis and hepatitis are most of the time missing. A probable malignant diagnosis was raised in 3 cases. Clinical outcome was protracted (15–35 days) but favorable.

To our knowledge, our study is the first pediatric case series of lymphoma-like syndrome. This clinical presentation is a source of delayed diagnosis due to diagnosis pitfall.

## INTRODUCTION

*Cytomegalovirus* (CMV) is a DNA virus belonging to the Herpesviridae family (*Human herpesvirus 5*), transmitted in blood, tissue transplantation, and also through saliva, genital secretions, urine, and breast feeding. After primary infection, latency is established, and reactivations can occur particularly in immunodeficient individuals. Primary CMV infection is common during early childhood: the prevalence rate, in industrialized countries, for children from 1 to 4 years old is estimated to be 43%.^1^

CMV infection and reactivation have been extensively explored in immunocompromised subjects because they can induce frequent and severe clinical manifestations.

In immunocompetent persons, primary CMV infection is self-limited infection, more often asymptomatic, and sometimes mononucleosis-like syndrome, meeting the Hoagland criteria of mononucleosis: fever, tonsillitis, lymphadenopathies, and lymphocytosis with ≥10% of atypical lymphocytes.^2^ However, atypical presentations have also been described in immunocompetent patients such as hepatitis, Guillain–Barré syndrome, colitis, myocarditis, pericarditis, thrombocytopenia, and autoimmune hemolytic anemia.^3^

Lymphoma-like syndromes associated with primary CMV infection have also been reported in adults.^4^ Prolonged febrile syndromes, alteration of the general status, large adenopathies, and hepatosplenomegaly are the hallmarks of this form of infection. To our knowledge, lymphoma-like syndrome revealing primary CMV infection has never been described in pediatric patients.

We report here 4 atypical primary CMV infections in immunocompetent children with lymphoma-like syndromes resulting in diagnostic difficulties necessitating multiple explorations.

## MATERIALS AND METHODS

Lymphoma-like syndromes were retrospectively reviewed in children attending the Montpellier University Hospital from January 1, 1999 to December 31, 2008 for primary CMV infection. Immunocompetent children ages 1 month to 18 years with lymphoma-like syndrome during CMV primary infection and without biological mononucleosis-like syndrome were included.

Definition of lymphoma-like syndrome was a protracted fever (≥15 days), an alteration of the general status (asthenia, anorexia, and weight loss), and a clinical lymphoproliferative syndrome defined by lymphadenopathies with malignancy criteria (firm, fixed, or supracentimetric) or hepatomegaly or splenomegaly. Diagnosis of primary CMV infection was based on serological testing with the detection of CMV-specific immunoglobulin (Ig) M associated with the presence of low-avidity CMV-specific IgG or evidence of IgG seroconversion.

Immunodeficient patients, patients with primary Epstein–Barr virus coinfection, toxoplasmosis, or mycobacterial infection and patients with biological criteria of mononucleosis-like syndrome defined as a strong increase of circulating mononuclear cells and >10% of activated lymphocytes were excluded.

The ethics committee was consulted (Comité de Protection des Personnes Sud Méditerranée IV, Dr Dubois), and as per their response, in our country, a retrospective study did not need an ethical approval.

## RESULTS

### Population

In our hospital laboratory, between January 1, 1999 to December 31, 2008, CMV serological testing was performed in 2151 children. Of these, 101 patients (4.70%) had CMV-specific IgM.

Of the CMV IgM-positive group, 33 (1.53%) were excluded because of immunodeficiency and 37 (1.72%) because of unconfirmed primary CMV infection diagnosis.

Primary CMV infection was confirmed in 31 immunocompetent patients (1.44%). Out of them, 4 (2 girls and 2 boys) patients met the criteria for lymphoma-like syndrome. Median age was 59 months (range 33-82). All of the patients were hospitalized.

### Symptoms and Clinical Examination

The main reason for consultation was protracted fever. The median duration of fever was 21.5 days (range 15–27). It was associated with chills and sweats in 2 cases. Patients were referred to the hospital after an average of 18 days with fever (range 13–27). Digestive symptoms were associated in 2 cases (Table 1).

**TABLE 1:**
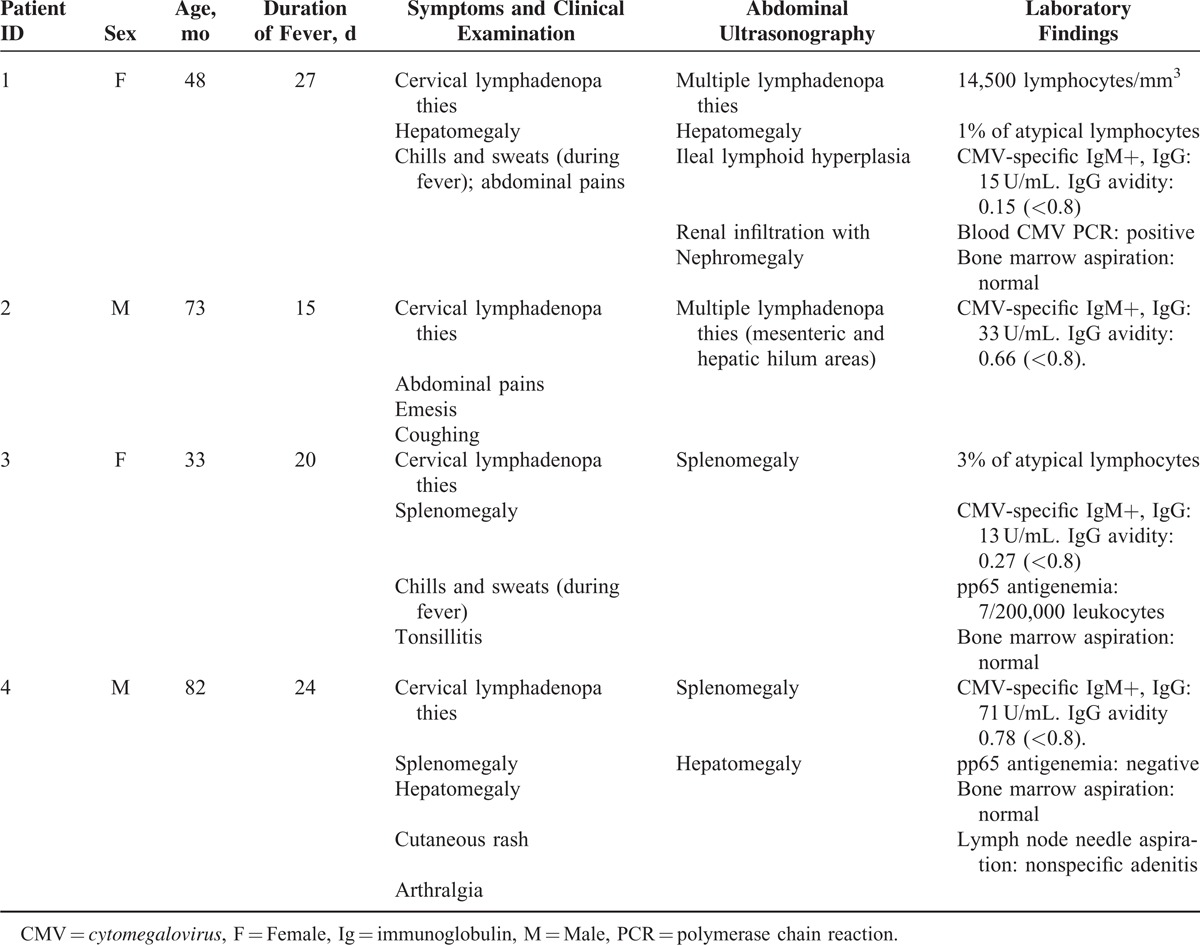
Characteristics of Patients With Lymphoma-Like Syndrome During Primary CMV Infection

Clinical examination showed hepatomegaly and/or splenomegaly in 3 cases and lymphadenopathies in all cases. Lymphadenopathies were cervical, suggesting a malignancy in 3 cases. Tonsillitis and cutaneous rash were rare (1 case each).

### Laboratory Findings

One patient had lymphocytosis (14,500 lymphocytes/mm^3^). Atypical lymphocytes in the leukocytic count were inconstant (2 cases), delayed, and in small proportion (1%–3% of lymphocytes). Cytopenia was not observed. One patient had a moderate thrombocytosis (518,000 platelets/mm^3^). C-reactive protein was moderately increased (mean 25 mg/L, range 10–37), and laboratory liver function tests (aspartate transaminase [AST], alanine transaminase [ALT], and γ-glutamyl transpeptidase) were within normal ranges.

### Medical Imaging

Abdominal ultrasonography was performed in all cases and confirmed clinical hepatomegaly and/or splenomegaly. Two patients had abdominal lymphoid hyperplasia: the first one had many lymphadenopathies in the mesenteric and the hepatic hilum areas. The second one had ileal lymphoid hyperplasia associated with many lymphadenopathies and renal infiltration with nephromegaly. Chest radiography was normal in all cases.

### Diagnostic Difficulties

Diagnosis of primary CMV infection was initially not suspected, and CMV serology was only performed between the 13th and the 29th day (mean, day 18) after the beginning of the fever.

A probable malignant diagnosis was raised in 3 cases. Bone marrow aspiration was performed and was normal. One patient had a lymph node needle aspiration, showing nonspecific adenitis. Neither adenopathy nor bone marrow biopsies were performed.

### Treatment and Outcomes

Two patients had only antipyretic treatment. The 2 other children were treated with antibiotics, macrolides, as a therapeutic test. Acetylsalicylic acid was prescribed for 1 patient with a suspicion of Still disease. Liver cytolysis without liver failure (AST 720 UI/L, ALT 2000 UI/L) occurred because of drug overdosage. Clinical outcome was good after stopping treatment. Corticotherapy was prescribed to this patient.

No patient received specific antiviral treatment.

Clinical outcome was protracted (15–35 days) but favorable for all patients. In the long term, no patient presented any sequel, and, to our knowledge, there was no diagnosis of malignant lymphoproliferative disorder or immune deficiency thereafter.

## DISCUSSION AND LITERATURE REVIEW

We describe 4 pediatric cases of lymphoma-like syndrome during primary CMV infection. Clinical presentation was protracted fever, alteration of the general status, and cervical lymphadenopathies associated in some of them with hepatomegaly and/or splenomegaly and/or abdominal lymphadenopathies. These clinical presentations induce diagnostic wavering due to the absence of tonsillitis and of biological mononucleosis syndrome.

To our knowledge, our study is the first pediatric case series of lymphoma-like syndrome during primary CMV infection. Only few adult cases have been described. Bonnet et al^4^ reported that among 115 hospitalized immunocompetent patients with primary CMV infection, 7% had pseudotumoral presentation (splenomegaly or lymphadenopathies with malignancy criteria) and 4% had lymphoma-like syndrome (alteration of the general status, protracted fever, lymphadenopathies with malignancy criteria, and hepatosplenomegaly). Complications described in that study were a subscapular splenic hematoma and a splenic rupture.^4^

In children, lymphadenopathies and hepatomegaly are more frequent during primary CMV infection,^5^ but lymphoma-like syndrome has never been described so far. The first reason is that there is no other large retrospective study about CMV primoinfection in immunocompetent children. The second reason is that lymphoma-like syndrome is very uncommon (only 4 children in 10 years in our hospital). However, among our 31 cases of primary CMV infection in immunocompetent children, lymphoma-like syndrome is one of the most frequent clinical presentation, and all the immunocompetent children with protracted fever (≥15 days) have a lymphoma-like syndrome (data not shown).

However, the retrospective feature of our study and potential selection bias related to the recruitment in a reference hospital may limit the generalizability of our observations.

## CONCLUSION

Pseudolymphoma syndromes during primary CMV infection have been poorly described, especially in children, and is probably underestimated. Protracted fever, alteration of the general status, and clinical lymphoproliferative syndrome are the key features of this syndrome that should justify a diagnostic CMV serology.

Lymphoma-like syndrome is a source of delayed diagnosis because many differential diagnoses exist and need to be eliminated. Although tonsillitis, hepatitis, and biological mononucleosis-like syndrome are missing, primary CMV infection must be raised as a surrogate diagnosis of malignant pathology.
